# Development of a Modular Research Platform to Create Medical Observational Studies for Mobile Devices

**DOI:** 10.2196/resprot.7705

**Published:** 2017-05-23

**Authors:** Martin Zens, Birgit Grotejohann, Adrian Tassoni, Fabian Duttenhoefer, Norbert P Südkamp, Philipp Niemeyer

**Affiliations:** ^1^ Medical Center - University of Freiburg Department of Orthopedics and Trauma Surgery Faculty of Medicine, University of Freiburg Freiburg Germany; ^2^ Medical Center - University of Freiburg Clinical Trials Unit Faculty of Medicine, University of Freiburg Freiburg Germany; ^3^ Medical Center - University of Freiburg Department of Oral and Maxillofacial Surgery Faculty of Medicine, University of Freiburg Freiburg Germany

**Keywords:** mHealth, telemedicine, mobile health, app-based study, research platform

## Abstract

**Background:**

Observational studies have proven to be a valuable resource in medical research, especially when performed on a large scale. Recently, mobile device-based observational studies have been discovered by an increasing number of researchers as a promising new source of information. However, the development and deployment of app-based studies is not trivial and requires profound programming skills.

**Objective:**

The aim of this project was to develop a modular online research platform that allows researchers to create medical studies for mobile devices without extensive programming skills.

**Methods:**

The platform approach for a modular research platform consists of three major components. A Web-based platform forms the researchers’ main workplace. This platform communicates via a shared database with a platform independent mobile app. Furthermore, a separate Web-based login platform for physicians and other health care professionals is outlined and completes the concept.

**Results:**

A prototype of the research platform has been developed and is currently in beta testing. Simple questionnaire studies can be created within minutes and published for testing purposes. Screenshots of an example study are provided, and the general working principle is displayed.

**Conclusions:**

In this project, we have created a basis for a novel research platform. The necessity and implications of a modular approach were displayed and an outline for future development given. International researchers are invited and encouraged to participate in this ongoing project

## Introduction

In recent years, mobile devices, such as smartphones and tablet computers, have become an important data acquisition tool in observational studies. Only in the last two decades have researchers noted a shift from traditional paper-based surveys to Web-based questionnaires [1-3]. The rapid spread of mobile phones and other devices, as well as the increasing functional abilities of those devices, has led to yet another shift in technologies that we are currently experiencing. An increasing number of medical professionals from various disciplines, for example, dermatology [4], psychology [5], gynecology [6], and pulmonology [7], are conducting studies based on mobile apps. While some reasons may be individually motivated, a number of advantages drive this development. In particular, “[t]hese research apps enhance widespread participation by removing geographical barriers to participation, provide novel ways to motivate healthy behaviors, facilitate high-frequency assessments, and enable more objective data collection” [8] by collecting the data in a domestic setting.

Piwek et al [9] have analyzed what prevents mobile phones from being the standard research tool for psychologists and conclude that “[s]martphones may only become an asset […] when development software that is both easy to use and secure becomes freely available.” In conclusion, the authors identified three reasons that limit the extensive growth and further use of mobile devices for research studies: (1) programming barriers, (2) consenting issues, and (3) concerns regarding privacy and data security.

The project presented in this paper depicts a Web-based platform solution aimed at solving these limiting factors with a clear focus on programming barriers and data security. The platform allows the creation of study apps without programming skills.

## Methods

**Figure 1:**
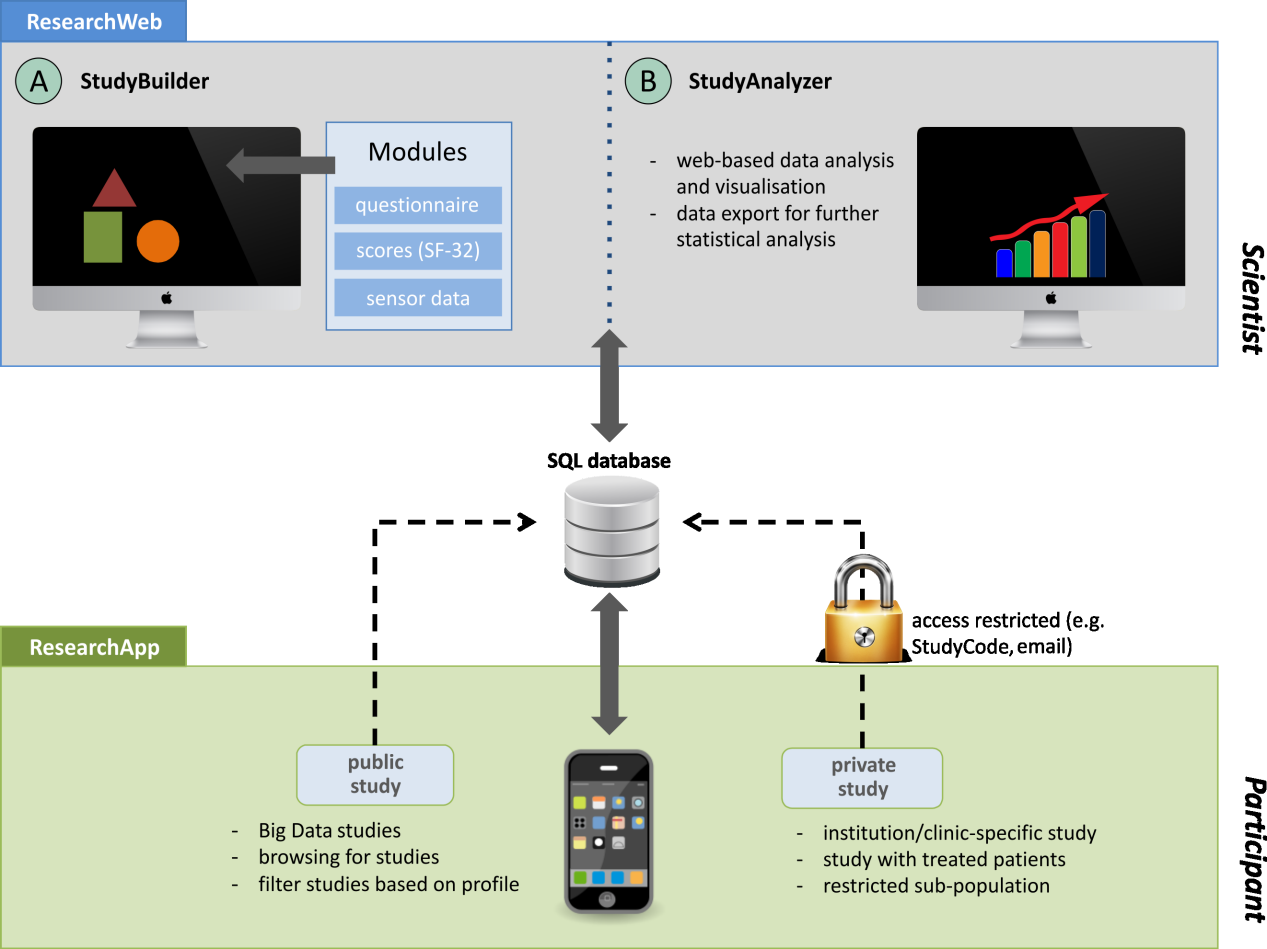
Schematic overview of the general concept.

The concept of our modular research platform focuses mainly on usability and an easy-to-use approach. A Web-based user interface to build studies (StudyBuilder) and to analyze ongoing studies (StudyAnalyzer) forms the key component of the platform. All created studies are stored in a database and become available via the study app (ResearchApp: ParticipantView). More complex studies, which exceed simple questionnaires to be answered by a participant, require the option to enter specific medical information by a medical professional. This feature is realized by a third essential component provided for doctors and other medical professionals (DoctorsView). Figure 1 gives a schematic overview of the general concept followed in this work without the DoctorsView.

**Figure 2:**
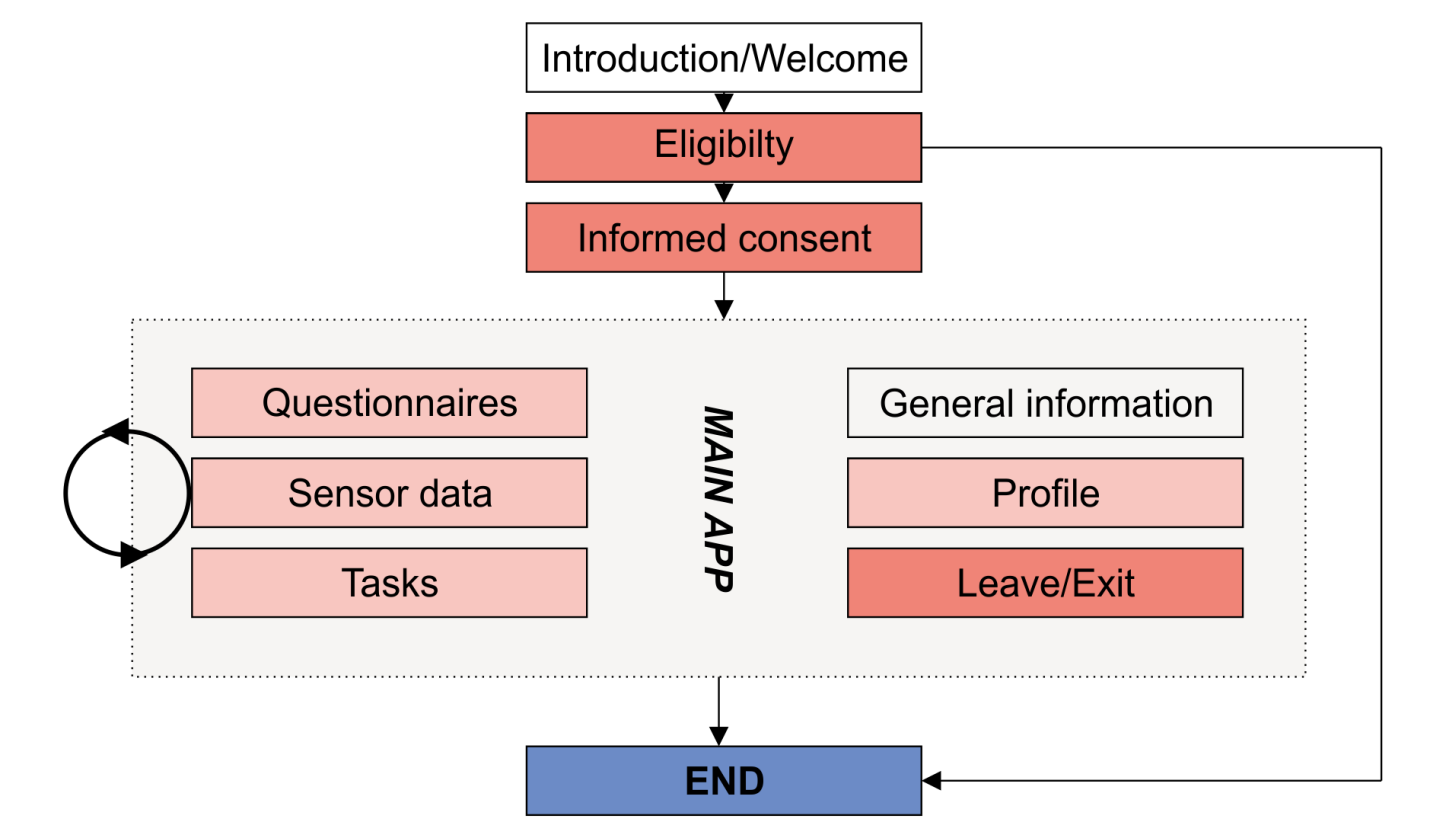
General concept of app-based observational studies. Red colored modules are mandatory.

In order to allow a flexible study design, a thorough analysis of essential components and required functions was necessary. Apple ResearchKit breaks down every observational app study into an introduction, an eligibility check, an informed consent, questionnaires, and surveys. Participant profiles, as well as sections with general information and progress of the study, are often included but not essential. Figure 2 depicts this concept [10-12].

### Module: Questionnaire

We further considered how app-based questionnaires are conducted and which functionality is needed. The required components can be broken down into different question types, transitions, and intervals. Question types can be open questions, multiple-choice questions, scale questions, and many more various kinds. In our terminology, transitions describe the flow within a study and intervals allow any sort of timing. All aspects are defined in more detail below.

Primarily, the concept of *modules* was introduced to manage studies. Each study consists of at least one or multiple modules (ie, questionnaires). Possible other modules are tasks, sensor data, reminders, and tips. In the current version of the research platform, only questionnaires are available and all further discussion in this work is limited to questionnaires. Every questionnaire consists of one or multiple questions. Each question is considered to be an *element* of the module.

Elements within a module and modules within a study are by default sequentially connected. When the last element of the last module is completed, the study is completed.

*Transitions* allow one to customize the flow of a study. A transition defines what is supposed to happen next, given external or internal conditions. A transition can define (1) a jump to a specific element (eg, question), (2) a jump to another module, (3) repetition of the same module, or (4) termination of the study.

The internal or external conditions can be determined simply by the outcome of one element (eg, answer to question 3 is yes), any numeric outcome (eg, age >30), a combination of numeric outcomes (eg, Comparative Pain Scale value 5 + continuous hours with this pain) or the overall outcome represented in a key figure out of multiple answers (eg, sum(), max(), avg(), min()). This idea also allows researchers to use established scores (eg, Short Form-36, Wells-Score, CHA_2_ DS_2_-VASc) to trigger transitions and influence the study flow.

In many studies, a timed execution of a module is desired. This may be regularly (eg, every 4 weeks) or with respect to a condition (eg, 6 weeks after surgery). The concept of *intervals* was introduced to (1) allow a timed execution of modules and (2) store answers and outcome of a module with respect to an interval.

By default, the study starts with the first module and ends with the last. The user can determine further starting modules that will also be triggered when the study begins, independent of any other events. This initiates a separate module flow.

### Module: Introduction, Eligibility Check, and Consent

Torous et al and Hwang et al [13,14] discussed the complexity of app-based eligibility checks and an informed consent. In summary, it can be stated that this issue is highly dependent on responsible ethics committees and applicable law in the country or region of the research institution. Again, the focus of our work is to provide tools within the research platform that facilitate principal investigators of a study adapting predefined solutions for their individual purposes. As concluded by Eysenbach et al [15], every medical study—app-based observational studies are no exception—require an eligibility check and an informed consent in order for participants to enroll. This project distinguishes between two main options: (1) a fully app-based information, eligibility check, and informed consent and (2) a paper-based enrollment by a doctor. The StudyBuilder provides an eligibility module that checks the inclusion criteria for the study and an informed consent module that allows a customized app-based consenting. A separate module is provided allowing a doctor to inform participants about a study and using the app only for support. This module is available only for closed, non-public studies. Different study types are as follows.

### Technical Architecture

The prerequisite for the concept presented in this paper is a centralized database structure. Data security and privacy are often mentioned as a disadvantage of mobile studies and reasons for researchers and participants to refrain from app-based studies [16,17].

Piwek et al [9] mention that data security is certainly not one of the strong sides of most medical professionals and thus requires the involvement of cost-intensive information technology specialists or computer science departments. A centralized data structure and shared research platform for numerous projects and research groups, as proposed in this work, eliminates the necessity to develop individual solutions regarding data security.

A structured query language database has been created that is stored on a webserver located in an independent German medical research foundation. Comprehensive German and European Union data protection law regulates the general conditions. High-end encryption algorithms have been implemented on the secure server, incremental backups are transferred to secure locations regularly, and all communications have been secured using secure sockets layer protocols. Most importantly, the established security level is continuously maintained and enhanced to meet new requirements and regulations.

### Customization, Language Support, and Study Publication

All studies can be customized and managed using the StudyBuilder. Customizing allows setting a study logo, colors, and institutional logos of the sponsor or research foundation. Currently English, French, Spanish, and German are the supported languages of the research platform. All studies may be created with single- or multilanguage support.

The StudyBuilder controls publication, pausing, and termination of studies. Each study may run in a predefined timeframe but may also be paused or terminated at any point by a (senior-) scientist. Studies may be published publicly or privately. Private studies are accessible only with an access code (StudyCode). This feature enables researchers to create studies for a predefined group of participants, for example, treated patients or within a specific institution. Public studies are available worldwide and may be accessed via the study browser in the participant app.

### User Concept, Profile, and Information

**Figure 3:**
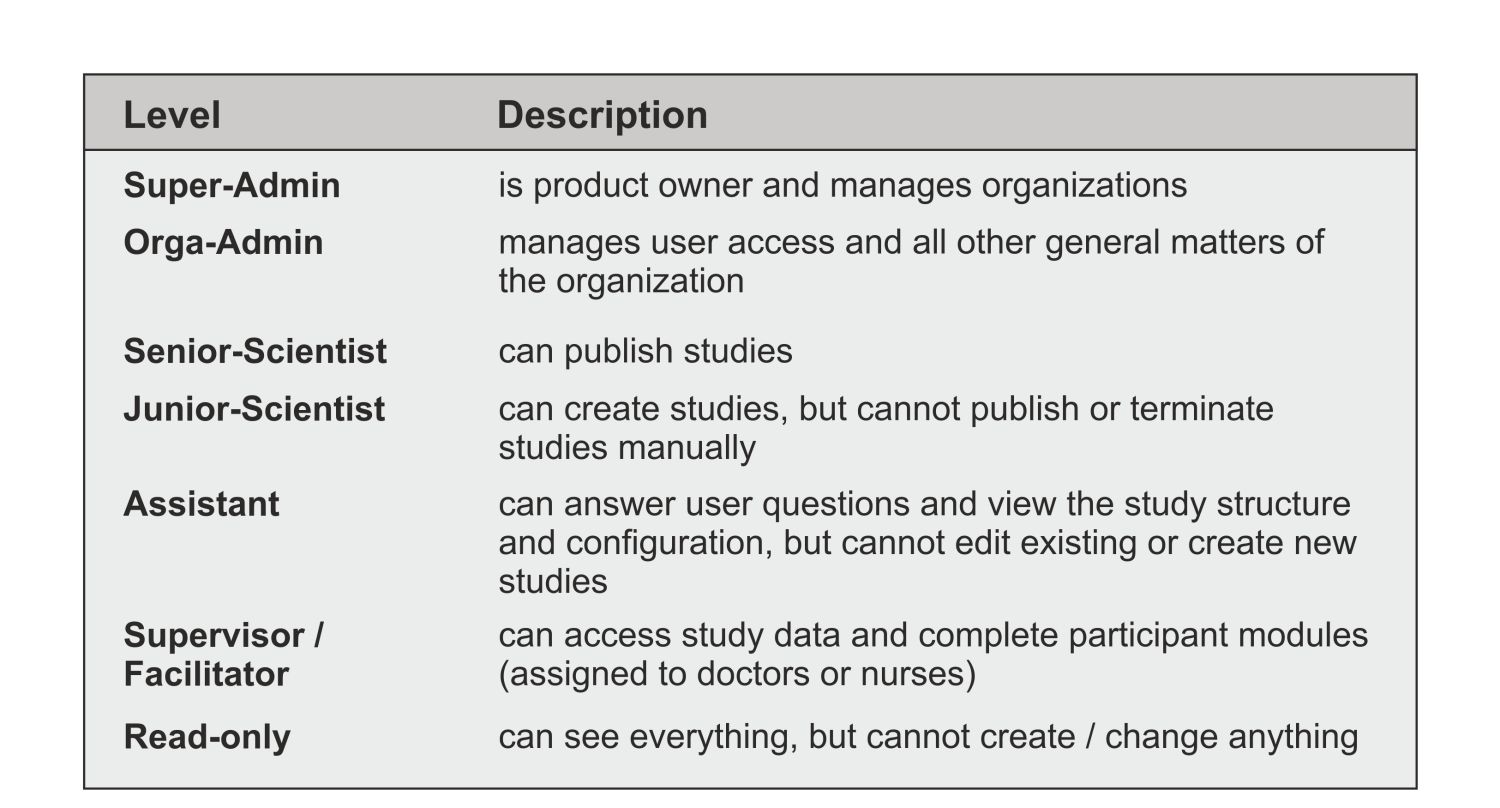
User concept for the Web-based part of the research platform (StudyBuilder and StudyAnalyzer).

Research is never a one-man show. Representing the complexity of research institutions in a Web-based research platform is challenging. We propose a solution with defined user roles for the Web-based research platform (Figure 3). Every user is assigned to an organization. Users may sign up to the research platform themselves or may be invited by email. The email contains a sign-up link. After completing the registration process, the user is either auto-verified, -approved, and -assigned by an institutional email address or they may request a new organization or enter an existing organization. A user may incorporate different roles in different studies.

**Figure 4:**
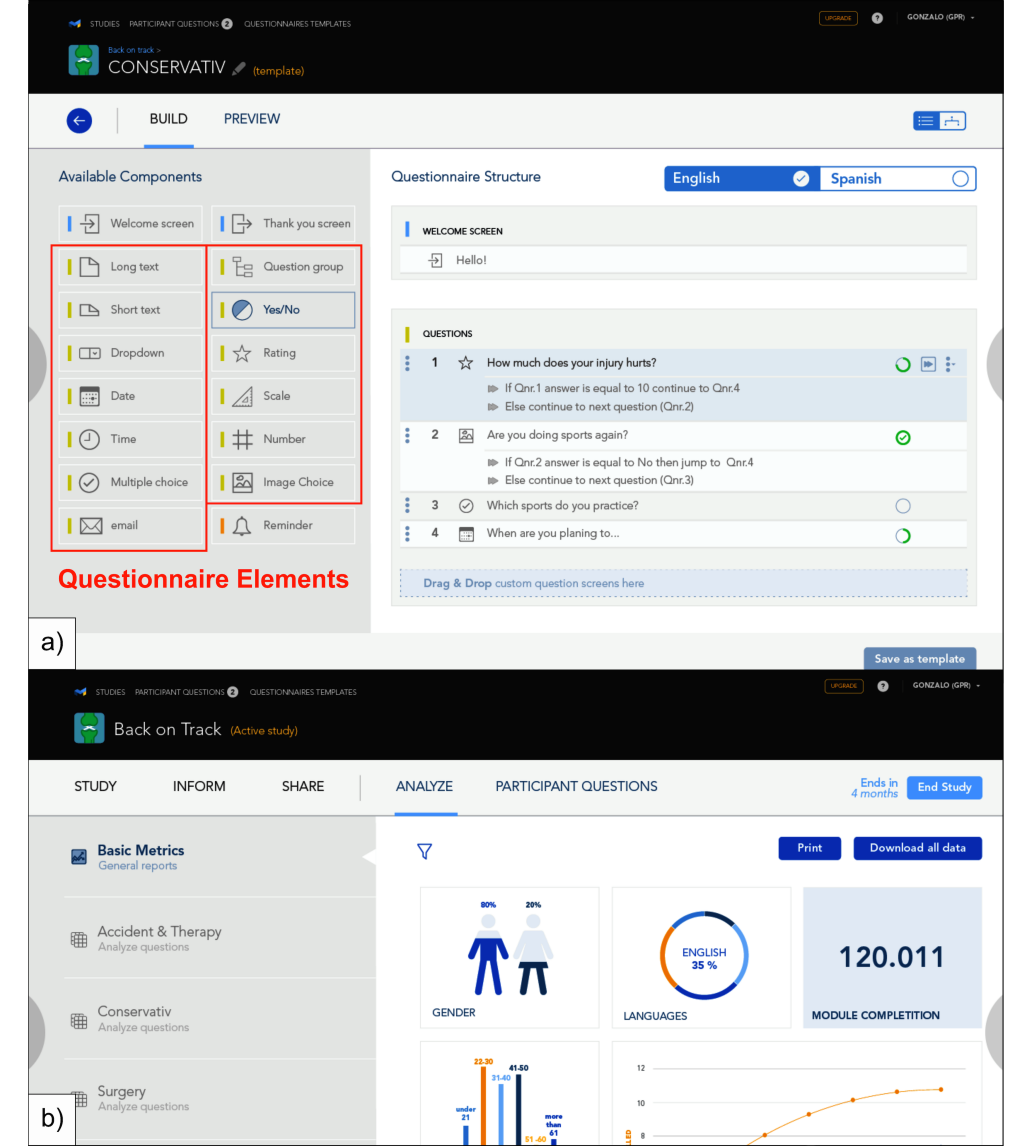
Screenshots of the current beta version of the a) StudyBuilder and b) StudyAnalyzer.

## Results

In January 2017, a beta version of the proposed research platform was completed and made available for a chosen group of testers. Figure 4 shows screenshots of the StudyBuilder and StoryAnalyzer. The StudyBuilder features multilanguage support and allows the composition of studies out of 13 prebuilt question types. In the current version, the StudyAnalyzer delivers basic demographic data on each study. A full dataset can be downloaded for further processing and statistics.

**Figure 5:**
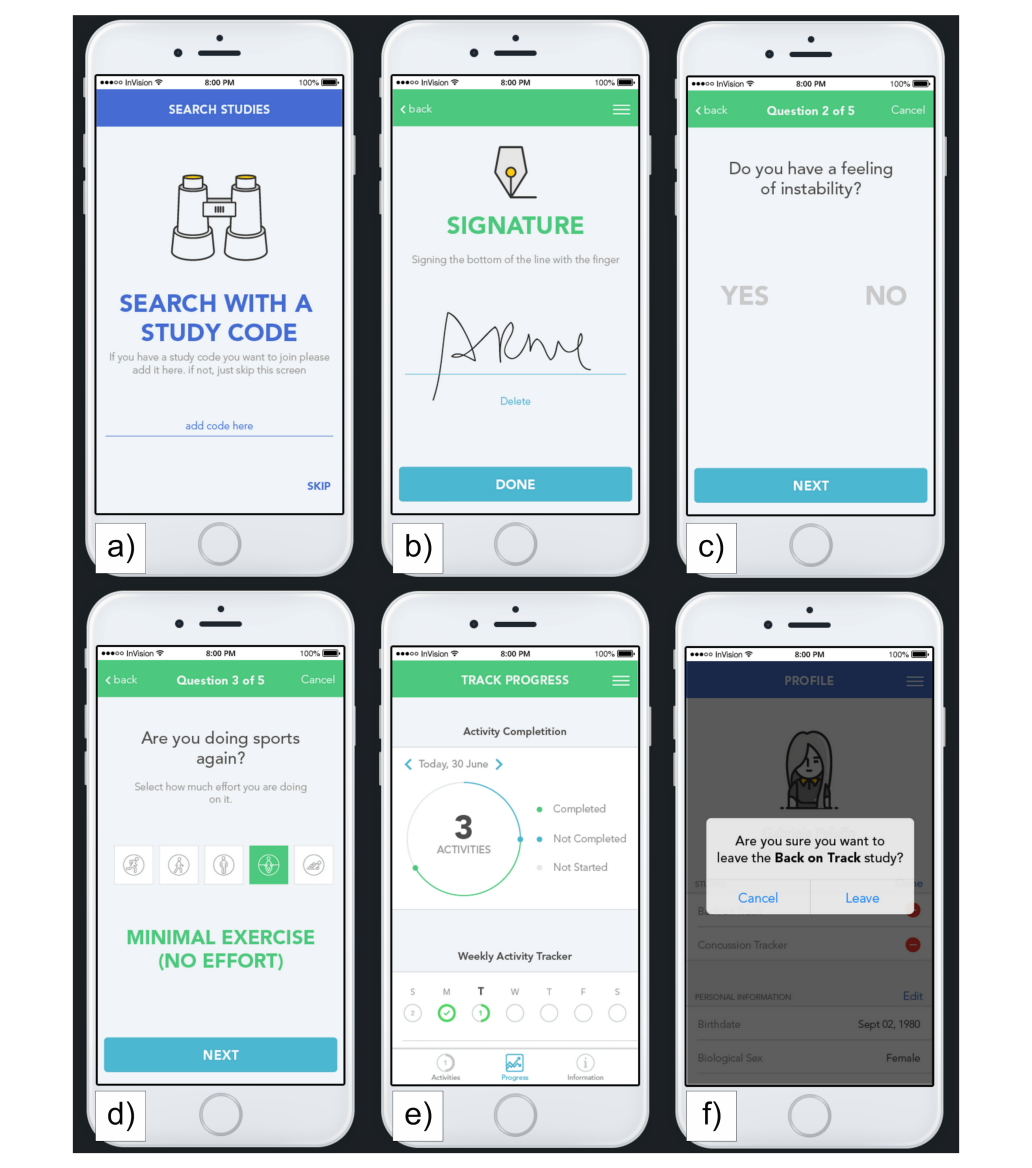
ParticipantView of an exemplary study created with the research platform.

Several steps of the ParticipantView of an example study are displayed in Figure 5. Each participant has to register to the research platform with basic demographic information. Then, every user can join one or multiple studies.

Within the first 12 months of this 24-month long project, we successfully developed a running prototype of a novel research platform to simplify app-based medical studies. The prototype consists of a strong database structure—a Web-based platform that can be used to easily create simple studies within minutes. Furthermore, it is possible to publish those studies for use in a study app. Studies can by multilingual and support complex user hierarchies.

## Discussion

### Principal Considerations

This work presents a novel research app development platform that allows the creation of mobile research apps without the need for programming skills.

In recent years, we have seen an increasing number of medical studies supported or entirely based on mobile apps. So far, little is known about the impact and merit of this technology, but first results are promising when this method is used adequately [8]. Specifically the psychology [18,19] and epidemiology [20] fields, but also every other medical discipline, are hoping to benefit from this technology and gain new insights regarding prevalence, diagnosis, and treatment of diseases [11,21].

Early studies, however, have identified challenges to this new technique, mainly programming and other technical issues [10,22]. In our opinion, it is neither cost-efficient nor time-efficient to develop individual apps for each study. The research platform we present lowers costs and improves quality as well as data safety. Apart from that, synergy effects in acquisition of participants are possible. A platform with possibly hundreds or thousands of studies might attract participants to join multiple studies or to take note of other studies and recommend those to family and friends.

The modular platform is itself built out of modules. Further functionality is planned and will be added throughout the entire first development period of 24 months.

### Comparison With Prior Work

In March 2015, Apple, Inc. announced the launch of ResearchKit, an open-source framework aimed at revolutionizing medical research studies. Until today, Apple is the only major software company providing such a framework [9]. During the initial presentation and announcement, the framework was described as a simple and easy-to-use tool for medical specialists. First studies [4,9,10,12,21] mutually agree that Apple has missed this goal of simplicity. Significant Object C or Swift programming skills are necessary to achieve a fully functional app.

Apart from that, ResearchKit supports only iOS devices. However, the majority of all mobile devices sold and being distributed is based on an Android system (Open Handset Alliance) [23]. This disadvantage has been recognized and addressed by two independent project groups. One of those being ResearchStack [24], which is maintained by Cornell Tech’s Small Data Lab and Open mHealth. A different solution called ResearchDroid [25] is commercially available and provided by Applied Informatics Inc.

ResearchStack is a fully functional software development kit and user experience framework aimed at developing research apps for Android devices. The framework is comparable to ResearchKit and aims at speeding up the transfer process for existing ResearchKit apps. This is achieved by “offer[ing] enough shared functionality and a common framework and naming scheme” [24].

ResearchDroid is “an Android library developed for automating survey forms and information consent building process” [25]. The library is very similar to the initial version of ResearchKit but allows the creation of Android instead of iOS apps.

**Figure 6:**
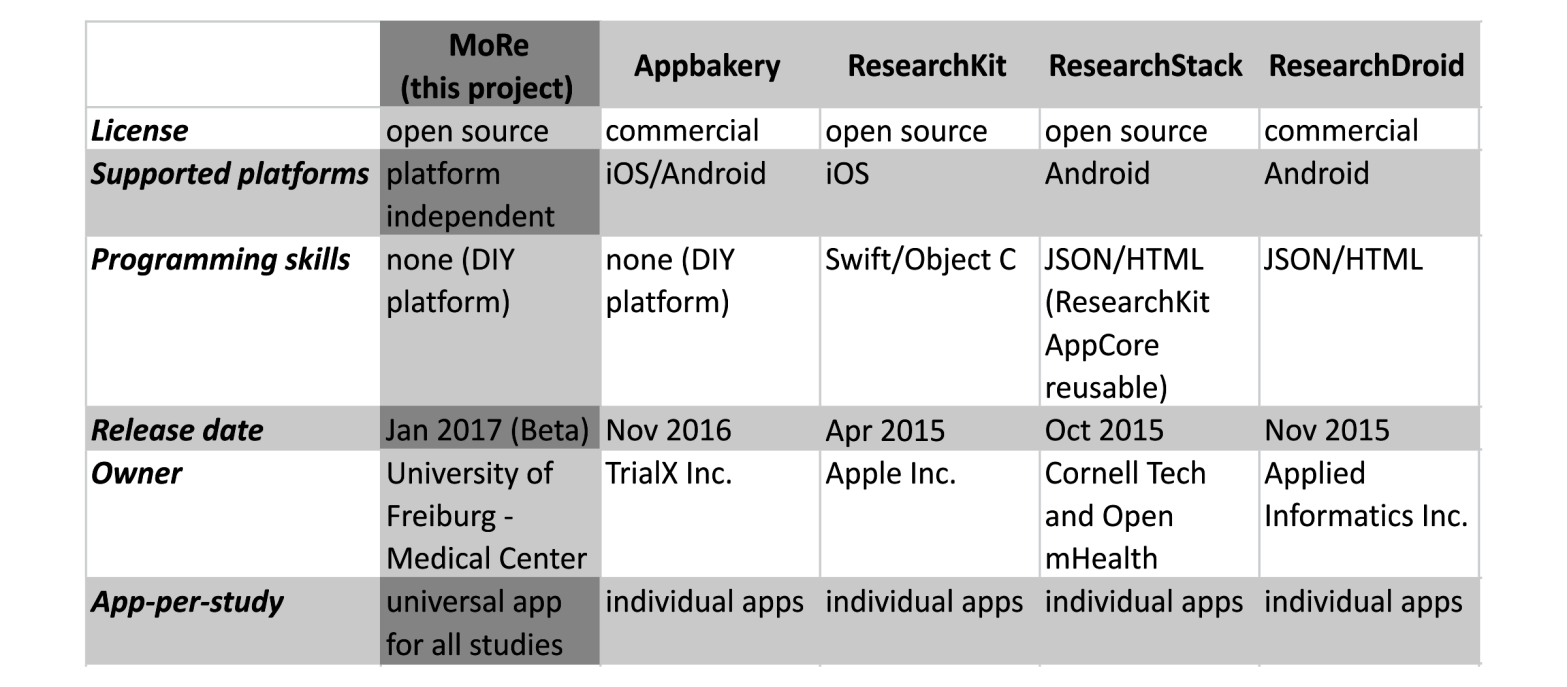
Comparison of currently available study building frameworks and platforms with the solution presented in this work.

ResearchKit, ResearchStack, and ResearchDroid have in common that all projects provide software libraries, frameworks, and development tools that require extensive programming skills to create apps. Appbakery (TrialX) [22,26], on the contrary, is the solution most similar to our work. The main goal of Appbakery as well as our approach is to enable researchers to create apps without programming skills. By using and integrating ResearchKit and ResearchDroid, Appbakery is capable of creating powerful and native iOS and Android apps. According to the company’s website, the product features HealthKit support, GoogleFit support, sensor support, and a data storage solution compliant with the Health Insurance Portability and Accountability Act, as well as simple surveys and prebuilt consent modules. A monthly fee is charged for this commercial solution. Figure 6 compares these different solutions.

To the best of our knowledge, other examples of comparable work exist only for computer-based software. Notable ones are PsychoPy [27] or LabView (National Instruments) for technical measurement systems. Both examples enable users to create individual software solutions with a graphical user interface engine and without programming skills.

### Limitations

A limitation of this work is that mainly only technical problems are addressed and potentially resolved. Social implications of a centralized research platform are yet unknown. Currently, critics argue that this technology has the possibility of security breaches. Furthermore, a vast percentage of the population is very sensitive and cautious about sharing personal health data via mobile devices. However, in our opinion this platform could also be a chance to build trust in this technology. With time and success, skepticism might vanish.

Another limitation is that not every study design can be created with the StudyBuilder. Although numerous options and possibilities have been taken into account, it is technically impossible to be prepared for every possible research scenario.

### Conclusions

This ongoing project attempts to solve many issues regarding mobile phone research. According to the United Kingdom’s National Health Service, development costs currently range from £1000 to £30,000 depending on the extent and functionality of a study app [11,28,29]. It seems necessary to concentrate resources for the development of a uniform and secure platform rather than supporting individual developments.

Furthermore, computer scientists, clinical doctors, psychologists, and many other professions are asked to work together for an all-embracing solution. The ultimate goal has to be a patient-oriented solution that is cost-effective, meets researchers’ needs, and helps gather important medical data for a broad variety of diseases.

The first 12 months of the project were used to develop a first fully functional version of the proposed research platform that allows the creation of simple survey-based studies. Sensor support, HealthKit and GoogleFit connectivity, automated trial registration, and extended backend functionalities will be added in the second project phase. Our institution has agreed to cover maintenance and support subsequent to the initial development phase of 24 months. Possible contributors and additional funding for further development are currently being identified. The vital source code will be available online under the Creative Commons license.

Scientists or companies willing to contribute to this project are welcome to contact the authors.

## References

[1] Best S, Krueger B, Hubbard C, Smith A (2001). An Assessment of the Generalizability of Internet Surveys. Social Science Computer Review.

[2] Bandilla W, Bosnjak M, Altdorfer P (2003). Survey Administration Effects?: A Comparison of Web-Based and Traditional Written Self-Administered Surveys Using the ISSP Environment Module. Social Science Computer Review.

[3] Braithwaite D, Emery J, De Lusignan S, Sutton S (2003). Using the Internet to conduct surveys of health professionals: a valid alternative?. Fam Pract.

[4] Webster DE, Suver C, Doerr M, Mounts E, Domenico L, Petrie T, Leachman SA, Trister AD, Bot BM (2017). The Mole Mapper Study, mobile phone skin imaging and melanoma risk data collected using ResearchKit. Sci Data.

[5] BinDhim NF, Alanazi EM, Aljadhey H, Basyouni MH, Kowalski SR, Pont LG, Shaman AM, Trevena L, Alhawassi TM (2016). Does a Mobile Phone Depression-Screening App Motivate Mobile Phone Users With High Depressive Symptoms to Seek a Health Care Professional's Help?. J Med Internet Res.

[6] Marko KI, Krapf JM, Meltzer AC, Oh J, Ganju N, Martinez AG, Sheth SG, Gaba ND (2016). Testing the Feasibility of Remote Patient Monitoring in Prenatal Care Using a Mobile App and Connected Devices: A Prospective Observational Trial. JMIR Res Protoc.

[7] Honkoop PJ, Simpson A, Bonini M, Snoeck-Stroband JB, Meah S, Fan CK, Usmani OS, Fowler S, Sont JK (2017). MyAirCoach: the use of home-monitoring and mHealth systems to predict deterioration in asthma control and the occurrence of asthma exacerbations; study protocol of an observational study. BMJ Open.

[8] Dorsey ER, Yvonne CY, McConnell MV, Shaw SY, Trister AD, Friend SH (2017). The Use of Smartphones for Health Research. Acad Med.

[9] Piwek L, Ellis DA (2016). Can Programming Frameworks Bring Smartphones into the Mainstream of Psychological Science?. Front Psychol.

[10] Zens M, Woias P, Suedkamp NP, Niemeyer P (2017). “Back on Track”: A Mobile App Observational Study Using Apple's ResearchKit Framework. JMIR Mhealth Uhealth.

[11] Jardine J, Fisher J, Carrick B (2015). Apple's ResearchKit: smart data collection for the smartphone era?. J R Soc Med.

[12] Chan YY, Wang P, Rogers L, Tignor N, Zweig M, Hershman SG, Genes N, Scott ER, Krock E, Badgeley M, Edgar R, Violante S, Wright R, Powell CA, Dudley JT, Schadt EE (2017). The Asthma Mobile Health Study, a large-scale clinical observational study using ResearchKit. Nat Biotechnol.

[13] Torous J, Roberts LW (2017). The Ethical Use of Mobile Health Technology in Clinical Psychiatry. J Nerv Ment Dis.

[14] Hwang M, Kwak IJ (2015). Description of a Mobile-based Electronic Informed Consent System Development. Stud Health Technol Inform.

[15] Eysenbach G, Wyatt J (2002). Using the Internet for surveys and health research. J Med Internet Res.

[16] Turner-McGrievy GM, Hales SB, Schoffman DE, Valafar H, Brazendale K, Weaver RG, Beets MW, Wirth MD, Shivappa N, Mandes T, Hébert JR, Wilcox S, Hester A, McGrievy MJ (2016). Choosing between responsive-design websites versus mobile apps for your mobile behavioral intervention: presenting four case studies. Transl Behav Med.

[17] Wazid M, Zeadally S, Das AK, Odelu V (2016). Analysis of Security Protocols for Mobile Healthcare. J Med Syst.

[18] Miller G (2012). The Smartphone Psychology Manifesto. Perspect Psychol Sci.

[19] Gan S, Goh B (2016). Editorial: A dearth of apps for psychology: the mind, the phone, and the battery. Sci Phone Appl Mob Devices.

[20] Marcano Belisario JS, Jamsek J, Huckvale K, O'Donoghue J, Morrison CP, Car J (2015). Comparison of self-administered survey questionnaire responses collected using mobile apps versus other methods. Cochrane Database Syst Rev.

[21] Bot BM, Suver C, Neto EC, Kellen M, Klein A, Bare C, Doerr M, Pratap A, Wilbanks J, Dorsey ER, Friend SH, Trister AD (2016). The mPower study, Parkinson disease mobile data collected using ResearchKit. Sci Data.

[22] TrialX Appbakery - Mobilize Research.

[23] Sui L (2016). Strategy Analytics: Android Captures Record 88 Percent Share of Global Smartphone Shipments in Q3 2016.

[24] Estrin D, Carroll M, Lakin N ResearchStack - An SDK for building research study apps on Android.

[25] (2016). ResearchDroid: An Android Forms and Consent Library.

[26] TrialX (2016). TrialX Launches AppBakery at AMIA - A DIY Researchkit App Platform Allowing Researchers to Build Cross-platform Study Apps without Developers.

[27] Peirce JW (2007). PsychoPy--Psychophysics software in Python. J Neurosci Methods.

[28] NHS Innovations South East (2015). NHS UK.

[29] Schweitzer J, Synowiec C (2012). The economics of eHealth and mHealth. J Health Commun.

